# Comment on a suite of mathematical solutions to describe ternary complex formation and their application to targeted protein degradation by heterobifunctional ligands

**DOI:** 10.1016/j.jbc.2021.100331

**Published:** 2021-03-01

**Authors:** Eugene F. Douglass, David A. Spiegel

**Affiliations:** 1 Department of Pharmaceutical and Biomedical Sciences, University of Georgia, Athens, Georgia, USA; 2 Department of Chemistry, Yale University, New Haven, Connecticut, USA; 3 Department of Pharmacology, Yale University, New Haven, Connecticut, USA

We are writing to express serious concerns about the recent paper published in JBC (https://doi.org/10.1074/jbc.RA120.014715) by Bomie Han entitled “A suite of mathematical solutions to describe ternary complex formation and their application to targeted protein degradation by heterobifunctional ligands.” (1) We believe that the author has incorrectly interpreted previous literature and misrepresented the scope and novelty of his contribution. While much of this work is novel and interesting, we believe claims of “universality” and “exactness” are misleading and critical mathematical and historical context is needed to frame this contribution appropriately.

Specifically, the author claims to have derived an “exact” solution to the cooperative ternary equilibrium, but has actually rederived an approximate solution, which was solved by Alan Perelson in 1980 (equation 33 in reference 2) (2). Indeed, our work from 2013 does provide exact mathematical solutions that describe both cooperative (equation 6 in reference 3) and noncooperative (equation 1 in reference 3) equilibria (3), but Han misstates this fact in his manuscript. In addition, several other equations—such as the equation for Emax—are presented as novel but were in fact first derived by Perelson in 1980 (equation 46 in reference 2) (2).

Critically, both Perelson’s and Han’s equations model a subset of ternary complex behavior represented by Quadrant 1 in Figure 4 of our published work (3). As a result, Han’s model is restricted to the subset of experimental conditions wherein [L]t = [L]; in other words, his findings are relevant only when the bifunctional ligand is in large excess over all other components. Obviously, this simplification does not always apply. Importantly, attempts by other researchers to employ Han’s models to systems that do not fulfill the [L]t = [L] approximation will lead to incorrect results.

Approximating the concentrations of free species to solve ternary complex equilibrium models has a rich history dating back to the 1950s as detailed by Segel (4). Proper historical context, therefore, is critical to establishing the novelty of this contribution. The author seems to overlook the seminal work of several other authors who have used similar free-concentration approximations to solve ternary complex equilibria, including:

• [L]t = [L] by Perelson (2)
• [P]t = [P] by Delean *et al.* (5)
• [P]t = [P] & [L]t = [L] by Hogg and Jackson (6)
• [P]t = [P] & [E]t = [E] by Segel (4)

Citing these precedents is further critical for establishing the utility of the mathematics, as each model is optimized for a specific experimental system (*e.g.*, DeLean’s model is optimal for modeling GCPR ternary complex signaling and is most utilized within that field (5)).

Overall, we believe that, in its current form, this manuscript is misleading and could hamper readers’ understanding of how bifunctional ligands, ternary complexes, and degraders behave. We believe these issues can be addressed by removing claims of universal scope and adding critical historical and mathematical context to appropriately frame the novelty and scope of this contribution.
